# Social inequalities in health-related quality of life among people aging with HIV/AIDS: the role of comorbidities and disease severity

**DOI:** 10.1007/s11136-020-02413-9

**Published:** 2020-01-07

**Authors:** Jochen Drewes, Jennifer Ebert, Phil C. Langer, Dieter Kleiber, Burkhard Gusy

**Affiliations:** 1 Public Health: Prevention and Psychosocial Health Research, Freie Universität Berlin, Habelschwerdter Allee 45, 14195 Berlin, Germany; 2 International Psychoanalytic University, Stromstr. 3b, 10555 Berlin, Germany

**Keywords:** HIV, Health-related quality of life, Socioeconomic status, Health inequalities, Disease severity

## Abstract

**Purpose:**

While socioeconomic inequalities in health-related quality of life are well documented in the scientific literature, research has neglected to look into the reasons for these inequalities. The purpose of this study is to determine in what way social inequalities in health-related quality of life among patients with the same chronic disease could be explained by variations in disease severity.

**Methods:**

We used the data of 748 people aging with HIV in Germany who took part in the nationwide study *50plushiv* and provided self-report data on socioeconomic status, health-related quality of life (SF-12) and various markers of disease severity (comorbidity, falls, late presentation and AIDS diagnosis). Regression analyses were applied to determine the impact of SES on HRQOL after adjusting for disease severity variables.

**Results:**

The mental and physical subscales of the SF-12, comorbidity burden and falls were significantly related to SES. SES explained 7% of the variance in PCS scores and 3% of the variance in MCS scores after adjusting for age and time since diagnosis. Markers of disease severity explained 33% of the variance in PCS scores and 14% of the variance in MCS scores. After adjusting for disease severity SES was still significantly related to PCS and MCS scores.

**Conclusions:**

The diverse sample of people aging with HIV showed social inequalities regarding HRQOL and most of the disease severity markers. SES was significantly related to mental and physical HRQOL after adjusting for disease severity. Possible explanations for this phenomenon are discussed.

## Introduction

Social inequalities in health-related quality of life (HRQOL) are well documented (e.g. [1–6]), but they are rarely in the focus of empirical studies [7]. In a recent paper, Mielck et al. demonstrated that educational attainment as an indicator of socioeconomic status (SES) is even among patients diagnosed with the same chronic disease significantly related to HRQOL, with patients from the lowest SES group assessing their HRQOL worse than patients from the higher SES groups [7]. Earlier studies show similar results for people living with HIV/AIDS [8], cancer patients [9] and patients with psoriasis [10], with other studies reporting non-significant results (see [7] for an overview). While we know that most chronic diseases exert a deteriorating effect on HRQOL [11], the question why the socioeconomic status of patients with the same chronic disease is related to HRQOL was never addressed before in the scientific literature.

Two factors that are known to correlate with HRQOL are disease severity [12–15] and comorbidity burden [16]. Studies also show that both are related to SES with patients from lower SES groups exhibiting higher levels of disease severity [17–19] and more comorbid diseases [20]. Insofar as disease severity and comorbidity burden are varying according to SES and are influencing HRQOL, these factors are likely explaining a great amount if not all of the social inequalities in HRQOL among people living with a chronic disease. Studies on these relationships between disease severity, comorbidity burden, socioeconomic status and HRQOL among patients with the same chronic disease are scarce and we could only detect one study on patients undergoing radical vasectomy after prostate cancer diagnosis. Here, to evaluate the differential impact of disease-related, personal and healthcare factors, as well as socioeconomic status on HRQOL in their sample, Klein et al. demonstrated that SES was still related to HRQOL ratings in various subdomains after controlling for disease severity markers, comorbidity burden and other factors [21].

People aging with HIV/AIDS, a subgroup of people living with HIV/AIDS (PLWH) that is growing in absolute and relative numbers since the advent of highly active antiretroviral treatment (HAART) [22], are facing huge challenges to the physical, mental and social aspects of their health. Comorbidities are widespread in this group [23], with the comorbidity burden being higher compared to the general population and younger PLWH [24]. Studies show that HRQOL is diminished for people living with HIV compared to the general population [25], but also compared to people living with other chronic diseases [26]. Aging people with HIV rate their HRQOL even worse compared to younger PLWH [27]. The comorbidity burden is significantly related to HRQOL in this group [23, 24] but we could not find any study looking into the influence of the socioeconomic status on HRQOL in this group.

The purpose of this study is to analyze the role of SES as a predictor of health outcomes and HRQOL among people aging with HIV/AIDS and to assess the unique impact of SES and disease severity and comorbidity burden to HRQOL to shed light on the question why HRQOL ratings are related to SES even among patients diagnosed with the same chronic disease.

## Methods

The study 50plushiv: psychosocial aspects of aging with HIV/AIDS in Germany’ was a cross-sectional, exploratory study with a quantitative and a qualitative study arm to describe the health, living conditions and needs of people aging with HIV and AIDS in Germany conducted in 2013–2014. Eligible participants were all people diagnosed with HIV, 50 years old or older (according to international research practices “older age” in the context of HIV is defined as 50 years and older e.g. [28]) and living in Germany.

The questionnaire was provided as an online questionnaire and as a paper pencil questionnaire. Several online and offline strategies were used to reach potential participants. Paper questionnaires were distributed amongst others via physician practices, hospitals and self-help organizations, the link to the online questionnaire was posted on various institutional social media profiles and integrated in their newsletters and media activities.

All information obtained from the participants is self-reported. All participants provided informed consent. The study was approved by the Freie Universität Berlin ethics committee.

### Sample

A total of 907 people who met the eligibility criteria completed either the paper (*n* = 499) or the online (*n* = 408) questionnaire. Due to missing data in the variables which we chose for the regression analyses, our final analysis sample consists of 748 participants. Our sample is a convenience sample which is not statistically representative of the population of people aging with HIV/AIDS in Germany.

### Measures

Besides sociodemographic and HIV-related variables that we only use to describe our sample, we chose eight variables for our regression analyses: age as a possible confounder variable, AIDS diagnosis, late presentation, physical comorbidities, psychiatric comorbidities and falls as markers of disease severity, socioeconomic status, and health-related quality of life as the dependent variable.

Late presentation: Late presentation with HIV was operationalized according to the consensus definition for “Presentation with advanced HIV disease” [29]. Participants were asked whether their CD4 count was below 200 cells/μL at the time of HIV diagnosis or whether they were diagnosed with an AIDS-defining illness at the time of HIV diagnosis. Participants were characterized as late presenters if they answered yes to any of these questions.

AIDS diagnosis: Participants were asked if they were ever diagnosed by a health professional with stage AIDS according to the Centers for Disease Control and Prevention’s classification of HIV infection [30].

Falls: Falls in older adults are considered a geriatric syndrome, i.e. a highly prevalent condition in older people that does not fit into discrete disease categories and has a substantial negative impact on quality of life and mortality [31]. Participants were asked if and how often they fell, stumbled or slipped without any evident reason in a way they lost balance and landed on the ground or a lower level in the preceding 12 months.

Physical comorbidities: We assessed the prevalence of 30 comorbid diseases and conditions relevant to PLWH according to our review of the literature and the input by experts from the field. We asked for the lifetime diagnosis of any of these physical comorbidities and whether the participant is currently in treatment for the condition or not. To group the diseases and conditions according to their impact on physical functioning we assessed beta-coefficients for each currently treated disease in a regression analysis with the physical component summary score of the SF-12 (see below) as the dependent variable controlling for age and time since HIV diagnosis. Using these beta-coefficients we grouped the diseases into three groups with a high (*β* > 0.2; 7 diseases), medium (*β* between 0.1 and 0.2; 12 diseases) or low (*β* < 0.1; 11 diseases) impact on physical functioning. See Table 2 for a list of the diseases and their impact on physical HRQOL. For each group we built an index by adding the currently treated diseases and conditions the participants reported.

Psychiatric comorbidities: Participants were asked if they were ever diagnosed with any kind of depression or any other psychiatric disorder by a health care professional and if they are currently in treatment for this disorder. For our analyses we calculated a dichotomous variable indicating whether the participant is currently treated for a psychiatric disorder or not.

Socioeconomic status: SES was assessed using a multi-dimensional aggregated index that was developed by Germany’s national public health institute for use in socio-epidemiological studies [32]. The index consists of the three-dimension education (i.e. school and professional education), occupation (i.e. professional status of the respondent or head of household) and income (i.e. net equivalent income). Scores for all dimensions are aggregated to a single SES score ranging from 1 to 7, with 1 being the lowest possible SES. Using data from a nationwide representative sample [32] we categorized our sample into three groups with a low (1st quintile in general population), middle (2nd to 4th quintile) and high SES (5th quintile). While we used these categories for descriptive analyses, we used the metric SES index for regression analyses.

Health-related quality of life: HRQOL was assessed using the German version of the generic SF-12 questionnaire, the short version of the SF-36 questionnaire [33]. Both scales are widely used to assess HRQOL in PLWH [34]. We chose the SF-12 because of its brevity and the existence of national norms. The SF-12 consists of 12 items and measures HRQOL on two dimensions, the physical component summary (PCS, 6 items, Cronbach’s *α* = 0.86 in our sample) and the mental component summary (MCS, 6 items, Cronbach’s *α* = 0.86) scores.

### Statistical analyses

Chi-square and t tests were used to compare health outcomes between SES groups. To analyze the relationships between SES, comorbidities, disease severity and HRQOL multiple linear regression analyses were employed with either the PCS score or the MCS score as the dependent variable. In model 1 we analyzed the impact of SES on HRQOL for both SF-12 subscales adjusted for age and time since diagnosis. In model 2, age and time since diagnosis (block 1), comorbidities and disease severity variables (block 2) and SES (block 3) were added blockwise to analyze the impact of SES after adjusting for comorbidities and disease severity.

## Results

### Sample characteristics

Table 1 shows sociodemographic and HIV-related characteristics of our analyses sample of 748 people aging with HIV/AIDS in Germany. The sample is predominantly male (87.2%) and between 50 and 59 years old (72.3%). Almost half of the participants reported having a higher education. Compared to the general population in Germany people with a lower SES (14.2% in our sample vs. 20% in the general population) and a middle SES (48.1 vs. 60%) were underrepresented in our sample while people with a higher SES were overrepresented (37.7 vs. 20%). Three quarters of our sample stated that they got infected with HIV through homosexual intercourse, the majority was diagnosed with HIV more than 10 years ago.

**Table 1:** Sociodemographic and HIV-specific characteristics of the sample (*n* = 748, Germany, 2013)

	%	*M* (SD)	Range
Gender			
Male	87.2		
Female	12.3		
Trans	.5		
Age		57.1 (6.5)	50–83
50–59 years	72.3		
60–69 years	21.1		
70 years and older	6.6		
Education			
Low	23.7		
Middle	29.7		
High	46.7		
Migration			
Yes	8.8		
No	91.2		
SES		12.5 (4.0)	3.3–21
Low	14.2		
Middle	48.1		
High	37.7		
Transmission group			
Homosexual transmission	75.9		
Heterosexual transmission	14.4		
iv drug use	3.5		
Blood products	4.1		
Other/don’t know	2.7		
Time since diagnosis		16.7 (8.5)	0–32
0–5 years	11.4		
6–10 years	17.8		
11–20 years	34.1		
More than 20 years	36.8		
Physical comorbidities		1.7 (2.2)	0–18
None	36.4		
1	23.8		
2	14.8		
3–4	16.5		
5 and more	8.4		

*SES* socioeconomic status, *iv* intravenous

### Comorbidities, disease severity variables and HRQOL stratified by SES group

Table 2 shows the prevalence for each of the 30 comorbid conditions and diseases and their impact on physical HRQOL after controlling for age and time since HIV diagnosis. Hypertension was by far the most frequently reported condition. Every third participant was currently treated for hypertension. Chronic pain, polyneuropathy and lung disease showed the strongest associations with the physical component of HRQOL.

**Table 2:** Prevalence of comorbid conditions and diseases the patient is currently treated for and their impact on physical HRQOL (*n* = 748, Germany, 2013)

	Prevalence (%)	*β*-coefficient
High impact comorbidities		
Chronic pain	16.4	− **.418**
Polyneuropathy	14.2	− **.292**
Lung disease (e.g. asthma, COPD)	12.2	− **.275**
Heart disease (e.g. heart failure, heart arrhythmia, valvular heart disease)	7.6	− **.234**
Rheumatism	5.2	− **.233**
Severe problems with attention/memory	5.0	− **.233**
Disease of the gastrointestinal system (e.g. peptic ulcer disease, inflammatory bowel disease, gastritis)	13.2	− **.215**
Medium impact comorbidities		
Hypertension	33.9	− **.191**
Hepatitis B	5.6	− **.182**
Kidney disease (e.g. kidney failure)	3.5	− **.177**
Osteoporosis	4.5	− **.165**
Hepatitis C	4.5	− **.165**
Peripheral artery occlusive disease	4.3	− **.159**
Myocardial infarction	4.7	− **.154**
Coronary artery disease	8.0	− **.153**
Diseases of the central nervous system (e.g. epilepsy, Parkinson's disease)	4.2	− **.145**
Stroke	3.2	− **.128**
Hodgkin lymphoma	.4	− **.113**
Lipodystrophy, lipoatrophy	3.8	− **.101**
Low impact comorbidities		
Diabetes mellitus	6.9	− **.093**
Cervical cancer	2.2^a^	− **.090**
Lung cancer	.3	− **.084**
Liver disease (e.g. liver cirrhosis)	6.6	− **.076**
Non-Hodgkin lymphoma	.3	− .064
Anal cancer	.9	− .037
Melanoma	.7	− .031
Colorectal cancer	.4	− .030
Kaposi’s sarcoma	.1	− .025
Prostate cancer	.5^b^	− .019
Breast cancer	3.3^a^	− .008

Coefficients in bold are significant at the .05 level. All regression models are adjusted for age and time since HIV diagnosis with the PCS scores of the SF-12 as the dependent variable

*COPD* chronic obstructive pulmonary disease

^a^Prevalence among female participants

^b^Prevalence among male participants

The distributions of the disease severity variables, comorbidities and the PCS and MCS subscales of the SF-12 for the total sample and stratified by SES group affiliation are shown in Table 3. The criteria for late presentation was met by almost a third of our sample, while the prevalence of late diagnoses was lower in the highest SES group compared to the lower SES groups, the effect was not significant. A third of our sample reported an AIDS diagnosis, with no significant differences between different SES groups. Reported falls were significantly related to SES in our sample. 17.2% in the total sample report a fall in the preceding 12 months, the prevalence in the highest SES group (11.7%) was almost half as high as the prevalence in both of the lower SES groups (20.8% and 20.6% resp.). With an average of 1.7 physical comorbid diseases and just 38% of our participants reporting no comorbidity they were currently undergoing treatment for (see Table 1), comorbidity was widespread in our sample. Every fifth participant reported being currently treated for a psychiatric condition. While participants from the lowest SES group reported more physical comorbidities than participants from higher SES groups for each impact index, only differences on the high impact and the low impact index are statistically significant. Differences in the prevalence of psychiatric comorbidities were statistically significant between SES groups: while 29.1% of participants in the lowest SES group were currently treated for a psychiatric disease, only 17.7% of participants in the highest SES group reported this.

**Table 3:** Health outcomes stratified by socioeconomic status (*n* = 748, Germany, 2013)

	SES low (*n* = 106)	SES middle (*n* = 360)	SES high (*n* = 282)	Total	Statistics
Percentage					
Late presentation	29.2	30.8	24.8	28.3	*χ*^2^ = 2.863 (*p* = .239)
AIDS diagnosis	35.8	34.2	33.3	34.1	*χ*^2^ = .219 (*p* = .896)
Falls	20.8	20.6	11.7	17.2	*χ*^2^ = 9.750 (*p* = .008)
Psychiatric comorbidity	29.1	21.9	17.7	21.4	*χ*^2^ = 6.202 (*p* = .045)
Mean (SD)					
Physical comorbidities					
High impact	1.1	.8	.5	.7	*F* = 11.521 (*p* = .000)
Medium impact	.8	.9	.7	.8	*F* = 1.657 (*p* = .191)
Low impact	.3	.2	.2	.2	*F* = 3.264 (*p* = .039)
HRQOL					
MCS	41.1 (12.2)	44.0 (12.3)	47.1 (10.8)	44.7 (11.9)	*F* = 11.256 (*p* = .000)
PCS	43.7 (10.7)	46.2 (10.5)	50.1 (9.0)	47.3 (10.3)	*F* = 20.291 (*p* = .000)

*SD* standard deviation, *HRQOL* health-related quality of life, *PCS* physical component summary SF-12, *MCS* mental component summary SF-12

Regarding HRQOL, both subscales showed a SES gradient with participants with a higher SES showing a 6-point higher average score on both scales than participants from the lowest SES group. While the sample average on the MCS subscale was 44.7, participants from the lowest SES group reached an average score of 41.1 and participants from the highest SES group had an average score of 47.1. Similarly, the average score on the PCS scale was 47.3 for the whole sample, while participants from the lowest SES group had an average score of 43.7 and participants from the highest SES group reached an average score of 50.1.

### SES, comorbidities, disease severity and HRQOL

Table 4 shows the results of the regression analyses. SES was significantly related to both the PCS and the MCS subscale of the SF-12 after adjusting for age and time of HIV diagnosis, while the relationship with the PCS score was stronger (*β* = 0.260) than with the MCS score (*β* = 0.169). SES accounts for 6.7% of the variance in PCS scores in this model and 2.8% of the variance in MCS scores. After entering disease factors in the regression model, these factors accounted for much more explained variance in the SF-12 subscales than SES. 32.7% of the variance in PCS scores were explained by disease severity and 13.8% of the variance in MCS scores. Late presentation and AIDS diagnoses were not significant predictors of neither the PCS nor the MCS subscales. While high impact physical comorbidities were the strongest predictors of PCS scores (*β* = − 0.378), medium impact comorbidities and psychiatric comorbidities were also significant predictors of PCS scores (*β* = − 0.071 and *β* = − 0.081). Falls also were a significant predictor of PCS scores (*β* = − 0.208). The strongest predictor of MCS scores was psychiatric comorbidities (*β* = − 0.295), falls and high impact physical comorbidities were also significantly related to MCS scores (*β* = − 0.112 and *β* = − 0.098). After entering disease severity factors into the regression analyses, SES was still significantly related to PCS (*β* = 0.149) and MCS (*β* = 0.119) and added a significant part to the explained variance in both scales. With 2.1%, SES explained a third of its original explained variance on PCS scores after adjusting for disease severity factors. For MCS scores, SES explained 1.3% of the variance, less than half of the variance SES originally explained in the first model before adjusting for disease severity and comorbidity burden.

**Table 4:** SES and comorbidity/disease severity variables as predictors of health-related quality of life subdomains: multivariate regression models (*n* = 748, Germany, 2013)

	PCS		MCS	
	*β*-coefficient	*R*^2^ (change in *R*^2^)	*β*-coefficient	*R*^2^ (change in *R*^2^)
Model 1				
SES	**.260**	.084 (**.067**)	**.169**	.077 (**.028**)
Model 2				
Disease factors (entered blockwise as block 2)		.345 (**.327**)		.185 (**.138**)
Late presentation	− .003		.011	
AIDS diagnosis	− .050		− .015	
PC high	− **.378**		− **.098**	
PC medium	− **.071**		.026	
PC low	− .002		.038	
Psychiatric comorbidities	− **.081**		− **.295**	
Falls	− **.208**		− **.112**	
SES (entered as block 3)	**.149**	.366 (**.021**)	**.119**	.198 (**.013**)

Coefficients in bold are significant at the .05 level. All models are adjusted for age and time since HIV diagnosis (entered as block 1)

*PCS* physical component summary SF-12, *MCS* mental component summary SF-12, *SES* socioeconomic status, *PC high* summary index of physical comorbidities with high impact, *PC medium* summary index of physical comorbidities with medium impact, *PC low* summary index of physical comorbidities with low impact

## Discussion

This is the first study focusing on the relationship between socioeconomic status and health-related quality of life among older people living with HIV/AIDS. We were able to obtain a large and diverse sample of people aging with HIV in Germany. Participants reported more than one comorbid diseases on average, with almost two thirds of the sample reporting a comorbid physical disease they were currently treated for and every fifth participant was currently treated for a psychiatric comorbid disease. Comorbidity burden as well as HRQOL ratings showed strong social inequalities across our multi-dimensional index of SES, with participants from the lowest SES group showing a significant higher comorbidity burden, higher risk for falls and impaired HRQOL compared to participants from the highest SES group. Comorbidity burden and disease severity variables accounted for a large part of variance in both SF-12 subdomains, but after controlling for them in addition to age and time since diagnosis SES was still significantly related to the physical as well as the mental dimension of HRQOL.

While interpreting comparisons regarding disease prevalences and HRQOL ratings across studies is difficult because of different research designs and sample compositions, it is noteworthy that our sample seems to show a relatively good health compared to other studies. Balderson et al. who assessed the prevalence of 21 physical comorbid conditions and diseases in a sample of 452 people aging with HIV in the US, reported an average of 3.8 comorbid conditions, with 94% living with at least one of the assessed conditions [23]. It is most likely, that these differences in comorbidity burden can be explained by our assessment of conditions that the participants is currently treated for, while Balderson et al. did not give any information on the time frame or other specifications used for their assessment. Still, the average physical HRQOL ratings in our sample does not seem to be declined compared to the German general population. Gandek et al. report an average PCS score of 47.7 for participants aged 45 to 64 years in a nationally representative sample, which is similar from our average rating of 47.3 [35]. In fact, our participants of the highest SES group rated their physical HRQOL better than the overall German population in this age group. The MCS score showed a different picture. The average score in our sample (44.7) and the average score in every SES group was substantially lower than the average in the 45–64 age group in the general population (52.2).

It seems that people aging with HIV show impaired HRQOL in the mental domain compared to the general population but in general no impaired HRQOL in the physical domain. However, the HRQOL and other health outcomes of people aging with HIV with a low SES is substantially impaired compared to the general population. Data to compare our low SES group with the corresponding SES group in the general population unfortunately does not exist.

Comorbidity burden and disease severity, though strongly related to SES and HRQOL, did not explain differences in HRQOL between SES groups entirely. Even after controlling for these variables SES was still significantly related to both domains of HRQOL in our sample. As other studies are affirming these results for different diseases while using different sets of disease severity variables [21, 36], other explanations must be taken into account for this unique impact of SES on HRQOL. Based on the scarce literature on this topic, we propose three possible explanations: (1) Operationalization: our operationalization of disease burden/severity might have been insufficient. Though we captured an extensive list of comorbidities and other markers of disease severity, it is possible that comorbidities or severity markers that we did not integrate in our questionnaire might have explained the remaining variance in HRQOL. HIV symptoms might be such an important marker of disease severity. Moreover, our reliance on self-report data may have led to systematic biases between SES groups. (2) Measurement errors: the remaining variance explained by SES might stem simply from measurement errors. In a rather methodological approach, systematic differences between (demographic) subgroups on HRQOL instruments are seen as a product of differential item functioning (DIF; e.g. [37]). DIF exists according to this perspective when an item or instrument is not invariant in different groups, i.e. persons from different groups who are on the same level of the underlying variable show different probabilities of answering an item. However, the DIF approach is not concerned with explaining group differences on the item or the instrument level, and runs the risk of underestimating or even completely neglecting meaningful differences between sociodemographic groups that stem from different disease burdens. (3) Systematic differences in appraisal processes: research regarding the response shift phenomena in HRQOL reminds us that HRQOL measurements are subjective appraisals reflecting individual internal standards, values and meanings of HRQOL [38]. While response shift theory was originally concerned with changes in HRQOL measurements over time and focuses especially on adaptation processes following disease episodes, Rapkin and Schwartz expanded the focus of response shift theory to a general model of HRQOL appraisal [39] with cognitive appraisal being fundamental for any HRQOL measurement [40]. Cognitive appraisal is highly subjective and subject to intra- and interindividual differences. While response shift and quality of life appraisal theory were to our knowledge never applied to explain group differences in HRQOL ratings because of its original interest in explaining intraindividual differences, it is reasonable to argue that SES group affiliation is systematically related to any or all of these cognitive appraisal processes. Members of a lower SES group could understand HRQOL questions in a different way, retrieve different relevant experiences, possess different internal standards or choose different targets of comparison, and finally evaluate their experiences differently. We did not find any published research that would confirm our proposition that SES group affiliation is related systematically to biases in the cognitive appraisal processes underlying HRQOL judgements. But there are indications that research into this could be worthwhile. Rapkin et al. who developed the Brief Appraisal Inventory, an instrument to measure appraisal processes in the context of HRQOL judgements, reported that two of the five dimensions of this instrument, namely ‘interpersonal and independence’ and ‘spiritual growth and altruism’, are significantly related to education as a marker of SES group affiliation [41]. From a stress research paradigm Almeida et al. could demonstrate that individuals with a lower educational background appraised daily stressors as posing a greater risk to their financial situations and their self-concept than individuals with a higher SES independently of the stressor and stressor severity [42].

Our study shows strengths and limitations. The main strength of our study is the use of a complex, multi-dimensional instrument to measure socioeconomic status. Our measure combines three dimensions of SES, income, education and occupational status, and was recommended for and widely used in socio-epidemiological studies in Germany. While studies on social inequalities in health are mainly relying on rather one of the three dimensions to operationalize SES or a proxy variable [43], our approach permits the comparability of study results, the population-based assignment of group membership and the use of SES as either an ordinal or metric variable in statistical analyses. While we were able to reach a diverse and relatively large sample of people aging with HIV/AIDS in Germany, our sample is biased regarding our main variable of interest. Compared to the distribution of our SES variable in the general German population, individuals with a low SES and to a smaller degree individuals with a medium SES are underrepresented in our study. This ‘middle class bias’ is frequently seen in convenience samples (e.g. [44]) and a result of the self-selection processes that were involved in the generation of these kind of samples. Due to the study design, we had to rely completely on self-report data, including the presence of comorbid diseases with the risk of systematic under- or over-reporting of diagnosed diseases. However, epidemiological studies have largely relied on self-reported data, and the congruence between medical records and patient’s self-report on diagnoses seems relatively high (e.g. [45]). Another limitation is the lack of inclusion of other disease severity variables such as HIV-related symptoms in our study. As our study used a cross-sectional design, causality between the predictors and the dependent variables in our regression models cannot be inferred, and it is possible that HRQOL especially, the MCS score were influencing disease severity ratings.

To explore the relationship between SES and HRQOL further, more research is needed that directly focusses on this relationship and potential moderator and mediator variables. Studies that use complex SES measures and thoroughly operationalize disease severity can help us to better understand the role disease severity plays in SES-related differences in HRQOL. While focusing on patient samples with different diseases will help us to better understand, how disease characteristics shape the relationship between SES and HRQOL and disease severity and thus help clarify inconsistent results regarding the relationship between SES and HRQOL. Moreover, we need research to better understand to what extent variables that might mediate HRQOL judgements such as coping styles, social support or health-related stigma also systematically vary with SES. Finally, we think it is worthwhile and would further our understanding of demographic group differences in HRQOL assessments to apply the theory of quality of life appraisals to explain these differences.
