# Correction to: Social inequalities in health‑related quality of life among people aging with HIV/AIDS: the role of comorbidities and disease severity

**DOI:** 10.1007/s11136-021-02854-w

**Published:** 2021-07-04

**Authors:** Jochen Drewes, Jennifer Ebert, Phil C. Langer, Dieter Kleiber, Burkhard Gusy

**Affiliations:** 1 Public Health: Prevention and Psychosocial Health Research, Freie Universität Berlin, Habelschwerdter Allee 45, 14195 Berlin, Germany; 2 International Psychoanalytic University, Stromstr. 3b, 10555 Berlin, Germany

## Correction to: Quality of Life Research (2020) 29:1549–1557 10.1007/s11136-020-02413-9

The article “Social inequalities in health‑related quality of life among people aging with HIV/AIDS: the role of comorbidities and disease severity”, written by Jochen Drewes, Jennifer Ebert, Phil C. Langer, Dieter Kleiber and Burkhard Gusy, was originally published Online First without Open Access. After publication in volume 29, issue 6, page 1549–1557 the author decided to opt for Open Choice and to make the article an Open Access publication. Therefore, the copyright of the article has been changed to © The Author(s) 2021 and the article is forthwith distributed under the terms of the Creative Commons Attribution 4.0 International License, which permits use, sharing, adaptation, distribution and reproduction in any medium or format, as long as you give appropriate credit to the original author(s) and the source, provide a link to the Creative Commons licence, and indicate if changes were made. The images or other third party material in this article are included in the article’s Creative Commons licence, unless indicated otherwise in a credit line to the material. If material is not included in the article’s Creative Commons licence and your intended use is not permitted by statutory regulation or exceeds the permitted use, you will need to obtain permission directly from the copyright holder. To view a copy of this licence, visit http://creativecommons.org/licenses/by/4.0.

The original article has been corrected.

